# Effects of HIS-based intervention on patient education process and patient satisfaction with nurses' education

**DOI:** 10.1186/s13104-022-06046-8

**Published:** 2022-06-03

**Authors:** Tahereh Toulabi, Fatemeh Mohammadipour

**Affiliations:** Social Determinants of Health Research Center, Lorestan University of Medical Sciences, Khorramabad, Iran

**Keywords:** Hospital Information System, Patient Education, Nursing Management

## Abstract

**Objective:**

This quasi-experimental study (before and after intervention) was designed to determine the impact of hospital information system-based intervention on the patient education process and patient satisfaction in cardiac and cardiac intensive care units.

**Results:**

Each nurse was observed at the time of patient education on average on eight shifts (total of 256 shifts), and at last 1350 computerized reports before and after the intervention were analyzed and 150 patient satisfaction with nurses' education questionnaires were completed before and after the intervention.

After the intervention, the patient education scores were significantly improved (p < 0.001). In addition, the results of a survey of patients about the level of satisfaction with the quality of patient education showed a significant increase compared to before the intervention (p < 0.001).

The ability to easily, completely, and quickly edit and record the provided education, eased the process of patient education and documentation.

## Introduction

Although nurses are aware of the importance and legal and ethical responsibilities of patient education, it seems that they are not yet fully embracing their true role [1]. A study on nurses’ performance showed that a significant amount of care (62%) had been missed and that in 80% of cases, patient education was reported as one of the six missed nursing care [2]. In particular, a study found that 73.8% of the discharged patients stated that they needed to receive information about one or more of their health-related areas [3]. Training courses, educational videos; web-based programs are among the strategies implemented to carry out patient education [4–7]. However, the outcome of many of these methods is still unknown [8] or has little effect [9].

Electronic hospital information systems (HIS) are developed to achieve various goals such as managing storage, retrieval, information analysis, and research facilitation. In the field of nursing, it is only used to keep nursing reports [10, 11] and its educational capabilities are not widely used [12, 13]. Considering the capabilities of HIS, it can be used to improve the quality of nursing care and emphasize in-service education for nurses in hospitals [14–16].

Several studies have shown that despite the proper entering of nursing reports into the HIS, nurses have neglected the recording of patient education and discharge plans [14, 15, 17]. The reason for such shortcomings can be attributed to a lack of support for some forms, as well as the lack of ability to register specialized nursing services [11, 12].

Furthermore, studies on nurses’ understanding of working with HIS have shown that nurses rely less on their memory when using electronic documents due to their care options [18, 19]. In other words, by making changes to the system, the standards of education are always in place and the nurse will be able to study them at the right time, and based on the survey form, determines the training required for the individual patient, and records the necessary training of patient within the shortest time. Whereas other methods only increase the nurse’s awareness and may not always be available and, most importantly, do not record the training provided to the patient in the nursing report [18]. On the other hand, nurses can play an important role in the future long-term plans such as sustainable development, yet their role in the 2025 map of the health of Iran Health Information Technology is not completely clear. There is a demand for further improvement in this regard [20].

Therefore, following the challenges such as inadequacy of the patient education role of the nurse, the lack of both quality and content of patient education documentation in HIS, and given the fact that using technology and easy access to information is one of the indexes of WHOin country ranking, the use of HIS as an electronic case infrastructure, and at the same time as an educational tool is preferable to other methods. So far, only a few studies have addressed the educational role of HIS [21–24]. Such studies are observational, and no change has so far been made in the optimization of HIS based on the findings of other studies. To achieve the goal of enhancing HIS to improve nursing care, further research is needed to determine the most important components of a successful system and prevent costly errors [25].

In a previous study [26], by HIS optimization, the mean of bedside nursing care, nurse-patient, and team relationship have been improved. The present study is an extension to previously published research. Therefore, the present study was conducted for the first time in Iran with to determine the impact of a HIS-based intervention on the implementation of the patient education process and patient satisfaction.

## Main text

### Methods

Since studies have shown the need for high cardiac care to promote HIS and cardiovascular patients’ need for education during hospitalization [27], cardiac and cardiac intensive care units were selected for this study.

A comprehensive literature review failed to identify an available measure that could be used to address the sample size calculation. The study population consisted of all nurses of the cardiac wards and CCU of Shahid Madani Heart Hospital who were studied using the whole enumerating method (N = 32) in the years 2017–2019.

According to Heshmati Nabavi et al. [28], 140 patients in two groups of before and after based on P1 = 0.1, P2 = 0.4 were estimated.

Inclusion criteria included clinical nurses’ employment during the study (8 months) in the cardiac or CCU wards, and the willingness to participate in the study. Consciousness, at least three days of hospitalization on the cardiac or CCU wards, and literacy were the inclusion criteria for the patient. Sampling was completed through nonrandom quota sampling.

**Fig. 1:**
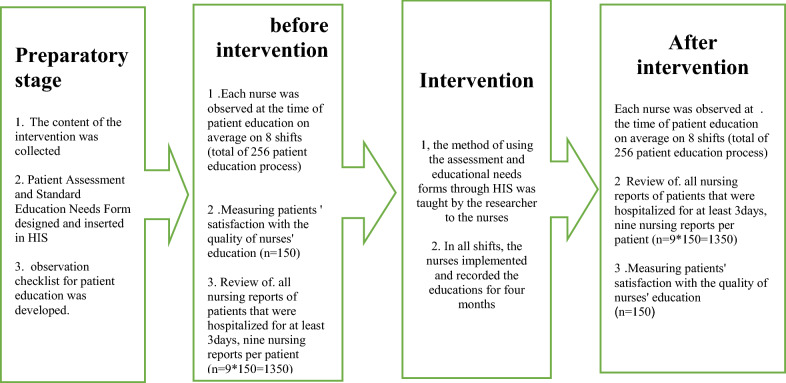
Details of the study steps

This research was a quasi-experimental study (before and after intervention) that was conducted in four stages: preparatory, before the intervention, intervention, and after intervention (for a better understanding of the study steps, please see Fig. 1).

#### Preparatory stage

In the pilot study, all the nurses, nursing managers, hospital managers, and IT unit tended to participate. At first, the goals and activities were examined. Based on the views of nursing managers and experienced CCU nurses, the Patient Assessment and Standard Education Need Form in collaboration with the HIS backing company (www.epd.ir/en) as an educational device was used to store or record information.

At this stage, the observation checklist for patient education was designed and installed in the HIS. The validity of the checklist was confirmed by the content validity method. This instrument was designed with 84 items and 8 axes: chest pain, rest-activity balance, cardiac patient diet, diabetic patient diet, nitroglycerine consumption, aspirin, constipation prevention, and participation in cardiac rehabilitation programs. The scores were allocated as: was done (2), was not recorded (1), was not done (0).

The internal reliability of the instrument was confirmed by the Cronbach alpha coefficient of 0.82. Furthermore, it was approved for sub-classes between 0.79–0.81.

Another tool was the Patient Satisfaction From Nurses' Education questionnaire has been designed by Heshmati Nabavi et al. [29]. This questionnaire comprised 12 close-ended statements (satisfied = 2, relatively satisfied = 1, and not satisfied = 0). Reliability of this self-report questionnaire by split half method and correlation coefficient, has been approved (0.85) [29]. The reliability of the questionnaire in the present study was ensured through α = 0.87.

#### Before intervention

Using information-gathering instruments, an assistant researcher observed the implementation of the patient education for two months; to minimize the effect of Hawthorne, she explained to the participants that the purpose of data collection was not to identify the errors in nurses’ work but understand the change in the process used to record patient education into an electronic system.

In addition, nine nursing records for 150 patients, registered in HIS (with emphasis on patient education) were reviewed before the intervention.

To collect patients' satisfaction with the quality of nurses' education, the researchers visited the wards during eight work shifts to select the participants. After determining the potential subjects, the authors briefed the patients or their family members and provided them with the questionnaire.

#### Intervention

At this stage, the method of using the assessment and educational needs forms through HIS was taught by the researcher to the nurses. The standard educational need included the title, objectives, training program, implementation, and evaluation that had been prepared in the system. The nurse read all the cases before education and after determining the educational needs based on the patient’s conditions, she/he gave the necessary education to the patient and then completed the implementation and evaluation sections based on the patient’s conditions and finally recorded in the patient’s computer file in the nursing report. In all shifts, the nurses implemented and recorded the education for four months. To facilitate the work processes, monitoring and formalizing the activities, the head nurse and educational supervisors, were selected as researchers’ partners and they continuously and daily supervised the training and gave feedback to the researcher. Meanwhile, the researcher appeared in various shifts to monitor and coordinate staff and respond to questions and receive feedback from nurses in the research environment.

#### After intervention

Within two months, the method of teaching patients was observed as intangible (controlling reports in HIS, visiting the patients) 8 times (based on the pilot) in different shifts by the researcher assistant. In addition, the records of 150 patients that were hospitalized for at least 3 days (nine records in the HIS) were reviewed. Also, during this period, data related to patients' satisfaction with nurse education were collected.

### Data analysis method

Data analysis was performed using descriptive statistics (absolute and relative frequency, mean and standard deviation), and inferential statistics (paired t-test or Wilcoxon) with *SPSS* 21.

## Results

### Nurses and patients' demographics

The mean and standard deviation of nurses’ age was 26.37 ± 4.72 years old. In addition, 87.5% were female, 59.4 were single.

Female patients constituted 55.3 and 51.4% of the sample group before and after the intervention respectively. The average age of the participants was 40.91 ± 18.03 and 42.8 ± 16.03 years before and after the intervention respectively.

### Patient education statistics

Paired T-test showed a significant increase (*p* = 0.001; *t* = 64.886) after the intervention. In addition, there was a significant difference between the mean and standard deviation of the patient’s education in the eight areas (Table 1).

**Table 1:** Patient education statistics

Education about:		*Mean*	*SD*	*Median*	*p*
Chest pain	Before After	4.28 7.09	1.08 1.32	4 7	0.001^b^
Rest-activity balance	Before After	0.59 6.22	0.61 1.36	1 6	0.001^b^
Cardiac patient diet	Before After	5.5 15.41	1.79 2.19	5 16	0.001^a^
Diabetic patient diet	Before After	5.34 25.47	1.59 1.45	5 25	0.001^b^
Nitroglycerine consumption	Before After	5.44 14.50	3.34 2.39	5 14	0.001^a^
Aspirin consumption	Before After	2.66 10.41	1.42 1.72	2 10	0.001^a^
Constipation prevention	Before After	1.63 12.22	1.21 1.18	2 12	0.001^a^
Cardiac rehabilitation programs	Before After	2.34 5.38	1.67 1.40	1 6	0.001^b^
Total	Before After	27.78 96.68	4.80 3.01	29.5 97	0.001^a^

^a^Paired Samples Test

^b^Wilcoxon Signed Ranks Test

### Patient satisfaction

After the changes, a 32% increase in satisfaction of cardiac patients was reported. In addition, according to Table 2, full satisfaction after changes with 85.7% has the highest frequency.

**Table 2:** Patients’ satisfaction of the patient education in cardiac and cardiac intensive care units

Satisfaction	Before *n* (%)	After *n* (%)	p
Satisfied (> 15)	7 10%	60 85.7%	< 0.001
Relatively satisfied (8–15)	52 74.3%	10 14.3%	
Not satisfied (< 8)	11 15.7%	0 0.0%	

## Discussion

The findings of the present study showed that patients' educational outcomes were improved after using the patient education reports in HIS. While most studies have focused on writing and documentation [25], no study has been conducted to optimize the system for use in the patient education process. In a descriptive-analytical study, Sheikhtaheri et al. found that facilitators' conditions positively direct their actual nurses' use of HIS. Information quality, service quality, and system quality significantly increase nurses' satisfaction with HIS. Lastly, nurses' satisfaction with HIS and their actual use positively improves their performance [30].

In the present study, consistent with the above findings, the existence of editable standards programs allowed nurses to acquire professional knowledge and then run it on a patient-specific basis, and record it for each individual in the shortest time.

Another important finding of this study was a significant increase in patient's satisfaction with nurse education. Current findings confirm similar work by Mills et al. that found high patients’ satisfaction with nurses’ use of point-of-care information technology in acute care. Congruent to our results, in a recent study, heart failure medication education resulted in improved patient-reported satisfaction scores and 30-day all-cause readmission rates [31] providing a training guide for patients, designing and installing a needs assessment and educational needs form in HIS will prepare the environment for HIS-based clinical education for clinical students and nurses, as well as for newly arrived clinicians. The ability for easy, complete, and quick editing and recording of the provided education helps in documentation thus this important, reliable, and accessible source can help conduct further research. Managers can also benefit from HIS by planning, organizing, upgrading manpower, controlling and evaluating, accrediting clinical governance, and implementing organizational excellence models this study has an important methodological point of view. Ahmadian et al. in their systematic review of 53 studies showed that no prior and subsequent studies have been conducted to evaluate HIS, which could be due to a lack of close collaboration between system developers and evaluators [25]. Kahouei et al. postulated that designing a study prior to and after the intervention method helps to determine how much the new system has solved the problem and helps investors to justify the costs and judgments about the possible costs of IT in the future [21].

## Conclusion

In this research, HIS was effective in the process of patient education. However, after more than a decade of using this system, it is time to provide users such as nurses with a new generation of HIS with a patient-centered approach and provide easy access to standards of care for implementation and assessment of patients.

### Limitation

One of the limitations of this study includes implementation in one educational hospital. It is recommended that in the future, research should focus on organizational strategies at the regional, national, and international levels and establish harmony among the strategic objectives of the organization, structure, organizational culture, and improvement of current processes.

## Data Availability

The data sets used and analyzed during this study can be provided from the corresponding author on reasonable request.

## Abbreviations

HIS: Hospital Information Systems
WHO: World Health Organization
CCU: Coronary Care Unit
